# The effect on blink frequency of a selective blue-filtering photochromic lens in patients with benign essential blepharospasm

**DOI:** 10.3389/fneur.2023.1301947

**Published:** 2023-12-18

**Authors:** Tobias Monschein, Tobias Zrzavy, Corinna Weber, Zoya Kuzmina, Walter Gutstein, Thomas Sycha

**Affiliations:** ^1^Department of Neurology, Medical University of Vienna, Vienna, Austria; ^2^Comprehensive Center for Clinical Neurosciences and Mental Health, Medical University of Vienna, Vienna, Austria; ^3^Austrian Healthcare Center, Vienna, Austria; ^4^OHI – Optometry & Hearing Aid Initiative, Vienna, Austria

**Keywords:** focal dystonia, blepharospasm, BEB, blue filter, lens

## Abstract

**Objective:**

The aim of this clinical study was to assess the impact of a non-invasive selective blue-filtering photochromic lens coating Crizal Prevencia on the treatment and response of benign essential blepharospasm (BEB).

**Patients and methods:**

Twenty-four patients were recruited in the outpatient clinic of the Medical University of Vienna in a randomized, double-blind, cross-over study design. Blink frequencies were assessed in patients with BEB before and 14 days after intervention with either a filtering ophthalmic lens or a placebo lens, respectively. Outcome parameters include sub-group analysis of a blink frequency under six different conditions: three photopic conditions, one resting condition, one reading condition, and one video game condition.

**Results:**

From 24 recruited patients, 15 patients were available for final analysis. Comparing the optical blue filtering lens to placebo, showed a reduced blink frequency in specific subtests, but not compared to baseline.

**Discussion:**

In conclusion, optical filtering glasses might have a beneficial effect on BEB and provide a non-invasive therapeutic add-on option, in addition to botulinum neurotoxin therapy, for patients with BEB and should necessarily be further investigated in a multicenter setting, resulting in larger sample sizes to gain valid information about the effect of photochromic blue filter glasses in BEB.

**Clinical trial registration**: https://drks.de/search/en/trial/DRKS00032135, DRKS00032135.

## Introduction

Benign essential blepharospasm (BEB) is the most common form of blepharospasm and is a focal form of dystonia characterized by excessive involuntary muscle contractions of the orbicularis oculi muscle (1). The prevalence reported is variable and approximately 3.6 out of 100,000 in Europe (2, 3).

It generally manifests in late adulthood, predominantly affecting women, and significantly impacts the quality of life of patients (4). While the underlying pathophysiology remains unelucidated, genetic and environmental factors have been repeatedly implicated (5).

In a study by Defazio et al., the following four diagnostic criteria for BEB were proposed and validated in a subsequent study with 211 BEB patients: (1) presence of stereotypical, bilateral, and synchronous orbicularis oculi muscle spasms; (2) effective sensory trick; (3) increased blinking; and (4) inability to voluntarily suppress the spasms (6, 7).

Local injections with botulinum neurotoxin (BoNT) into the orbicularis oculi muscle are considered a first-line treatment, and currently, the most effective treatment option for BEB patients (8). BoNT is required to be reinjected approximately every 2 to 3 months, and the most common reported side effects include ptosis, diplopia, dry eyes, and loss of efficacy during long-term use (9). Oral pharmacotherapy includes compounds targeting monoamines, acetylcholine, or gamma-aminobutyric acid, and is reported to have a potential additive effect on alleviating symptoms but is mostly inadequate as a stand-alone therapy.

The observation that BEB can be triggered and exacerbated by bright light, which can also be exemplified by the fact that patients were wearing sunglasses even on days with normal or low-light conditions, led to the investigation into photochromatic modulation as a potential treatment option for alleviating BEB-associated light sensitivity and severity (10, 11).

Initial studies using an FL-41 tinted lens showed improved light sensitivity in patients with BEB compared to gray lenses, and also showed improvement in symptom severity, including blink frequency, light sensitivity, and functional limitations (12, 13).

While the initial studies of migraine headache investigating intense orange-tinted lenses FL-41 show symptom alleviation, the absorptive tinted lenses are not suitable for continuous wearing since color vision, scotopic vision, and chronobiology rhythms may be significantly impacted. Moreover, the intense orange tint highly limits the aesthetics of the lens.

Here, we aimed to test in a randomized, double-blind, cross-over interventional study if a selective blue-filtering lens developed by Essilor, *Crizal*^**®**^ Prevencia™, which is constituted by an innovative interference optical filter coated onto an ophthalmic lens, provides symptomatic alleviation in BEB patients.

## Methods

### Patient selection

Eligible patients were screened in the outpatient clinic of the Department of Neurology, Medical University of Vienna, between April 2018 and August 2019, and patients fulfilling inclusion criteria were scheduled for a baseline visit (in the case of BoNT therapy, at least 12 weeks after patients received their last BoNT injection).

#### Inclusion criteria

Patients with idiopathic benign essential blepharospasm.

#### Exclusion criteria

Patients having any other disease with involuntary blinking (hemifacial spasm, tic disorder, and tardive dyskinesia); in case of an ongoing BoNT therapy <12 weeks since last BoNT injection, acute or chronic severe medical conditions that would preclude the subject from participating; patients with medical history of dementia or seizure disorder; those who were pregnant; those aged less than 18 years; and those who were unable or unwilling to give informed consent/to cooperate with study requirements.

During each study visit, inclusion and exclusion criteria were re-visited and reviewed for whether any new exclusion criteria arose since the former visit/initial screening.

### Trial design

This randomized, double-blind, cross-over study investigated the effect of photo-protective and photo-selective blue-filtering ophthalmic lenses on blink frequency in patients with BEB. After screening and patients signing consent, three study visits every 14 days were conducted: baseline, visit_14, and visit_28. Patients were randomized to group A (Crizal Prevencia = > Crizal Forte UV) or group B (Crizal Forte UV = > Crizal Prevencia) at the baseline visit. At visit_14, participants switched glasses according to their group assignment. All patients were tested at baseline, visit_14, and visit_28.

### Outcome measurements

The primary outcome measure is blink frequency under different conditions (measured manually by two independent raters) after wearing the blue-filtering ophthalmic lens (Crizal Prevencia) for 2 weeks compared to a placebo lens (Crizal Forte UV).

- The analysis of blink frequency was as follows:
  - resting condition (looking straight ahead)—3 min
  - reading a standardized text—3 min
  - playing a monitor interactive game—3 min
  - three different photopic conditions (99, 64, and 33% contrast)—each 6 min.

The order of the tests was as follows: first photopic test (99%), resting test, second photopic test (64%), reading test, third photopic test (33%), and video game testing condition.

### Lenses

#### Crizal Prevencia

The tested medical device is the Essilor lens ***Crizal***^**®**^ Prevencia™, the first photo-protective, and photo-selective blue-filtering ophthalmic lens. The specifications are defined as follows:

- Reflect a significant proportion of blue-violet light between 415 nm and 455 nm
- Limit the UV reflection on the backside
- Maximize the transmittance of blue-turquoise light between 465 nm and 495 nm
- Optimize the average visible transmittance and ensure the best anti-reflective efficacy.

**Figure 1:**
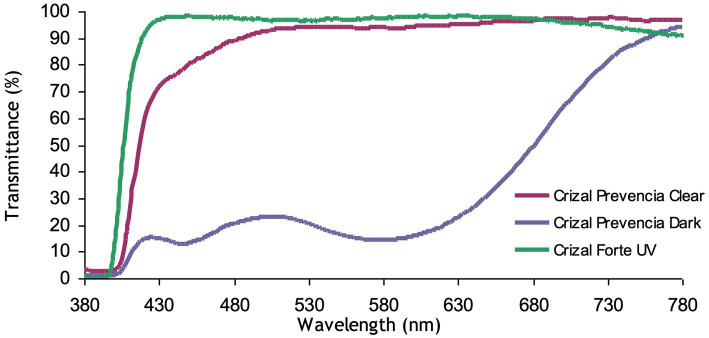
Transmittance of photochromic gray Crizal Prevencia vs. Crizal Forte UV on the MR8 substrate.

For the whole photochromic Crizal Prevencia lens,

- In a clear state, the harmful blue-violet is reduced by more than 30% for ultra-high indexes due to the interference filtering of the front side (~ 20%) plus the additional absorption of photochromic dyes (Figure 1)
- In a dark state, as photochromic lenses are tinted, the harmful blue-violet light protection is at its best, over 80%, whatever the substrate (Figure 1).

As required, the reflectance of beneficial blue-turquoise light is very limited, less than 2%.

#### Crizal Forte UV

The clear lens Crizal Forte UV is our placebo lens: no photochromic treatment and no blue-violet filtering. The total transmittance of the lens is represented in Figure 1.

### Statistical analysis

Statistical analysis was performed using R studio (Version 1.2.5033, RStudio, Inc.). Statistical significance was set at a two-sided *p*-value of 0.05 and is provided up to 0.001.

Normal distribution was assessed using Shapiro–Wilk’s method. As appropriate, group differences were assessed using a paired *t*-test or Wilcoxon signed-rank test. Due to the explanatory nature of the study, no post-hoc correction for multiple testing was performed.

For two patients, no values were available for the video game test at baseline. After sensitivity analysis, the imputation of missing data was handled using a missing at random (MAR) approach by multivariate imputation by chained equations using classification and regression trees.

Based on the study from Blackburn et al., we hypothesize a 30% reduction of blink frequency with the blue-filtering lens after wearing it for 2 weeks (13). The null hypothesis assumes that there is no difference in blink frequency between the blue-filtering lens and the placebo lens. For the calculation of the effect size, we defined the mean values μ_1_ = 1 (placebo) and μ_2_ = 0,7 (Crizal Prevencia).

Therefore, a sample size of 23 in a cross-over design with two study arms will have 80% power to detect an effect size of 0.86 using a paired *t*-test with a 0.05 two-sided significance level for the comparison of the effect of the blue-filtering lens to the placebo lens. To account for a potential study dropout, we therefore proposed a sample size of 30 to be included in this trial. However, the study had to be ended after 24 patients were included due to the low number of patients with BEB in the outpatient clinic.

### Approval, registrations, and patient consents

This study was approved by the Ethics Committee of the Medical University of Vienna (EK Nr: 1657/2014). We affirm that all methods described in this study were carried out in strict accordance with the relevant guidelines and regulations (EK Nr: 1657/2014). We confirm that informed consent was obtained from all prior to their participation in the study.

In addition, the study was registered in the German Clinical Trials Register (GermanCTR) on 3 July 2023. The corresponding registration number is DRKS-ID: DRKS00032135, and it can be accessed via the following link https://drks.de/search/en/trial/DRKS00032135.

## Results

Twenty-four subjects initially consented to participate. Nine participants dropped out of the study due to loss to follow-up (*n* = 4), incomplete data collection (*n* = 3), and repeated BoNT injection (*n* = 2). The cohort of dropped-out participants was significantly older than the participants completing the trial (61.81 vs. 71.34 years; *p* = 0.0072).

The final cohort for analysis consisted of 15 study participants (Table 1). Ten subjects were women, participants had a mean disease duration of 6.8 years (SD 7.76), two participants had a positive family history of blepharospasmus, and two-thirds received botox treatment, prior to study enrollment.

**Table 1:** Patient characteristics.

	*N* = 15
Age	61.81 (10.01)
Sex	
F	10/15 (67%)
Years with blepharospasmus, mean	6.80 (7.76)
Botox Tx:	10/15 (67%)
Diabetes	1/15 (6.7%)
CV disease: heart disease + hypertension	8/15 (53%)
Thyroid disease	4/15 (27%)
Hypertension	6/15 (40%)
Cancer	2/15 (13%)
Eyelid_lift	3/15 (20%)
Tobacco	3/15 (20%)
Alcohol	3/15 (20%)
FHx positive	2/15 (13%)

Age in years; FHx, family history positive for blepharospasmus.

According to the primary endpoint of the study, when comparing baseline blink frequency to verum or placebo, no significant difference in any of the six investigated conditions was observed.

**Figure 2:**
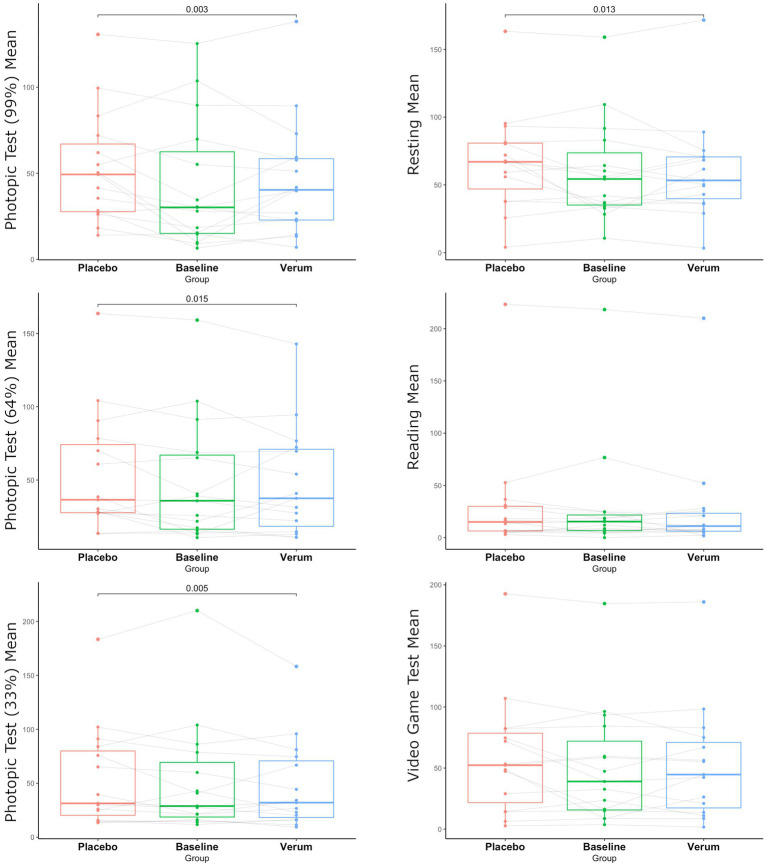
Comparing groups among different conditions.

Comparing the verum to the placebo group, significantly reduced blink frequency was observed in the verum group in conditions with 99, 64, and 33% contrast and under resting conditions (*p* = 0.003, *p* = 0.013, *p* = 0.015, *p* = 0.003, respectively) (see Figure 2).

## Discussion

Benign essential blepharospasm (BEB) is the most common form of blepharospasm and is a focal form of dystonia that causes a significant impact on the quality of life in individuals.

Light-induced triggering of BEB and photophobia, in general, has been shown to impact patients’ quality of life (14). While initial studies using the FL-41 tinted lense or 480 nm and 590 nm blocking lenses showed improved light sensitivity in patients, in this randomized, double-blind, cross-over study consisting of a small cohort of 15 patients, we tested the ability of a new selective blue-filtering ophthalmic lens (Crizal^®^ Prevencia™) in improving the blink frequency of BEB patients (13, 15).

While we found a significant reduction in blink frequency when using the Crizal^®^ Prevencia™ compared to a clear lens (Crizal Forte UV), there was no improvement in comparison to the baseline assessment. The lack of improvement in the verum group compared to the baseline cannot be explained based on the available data. A large-scale multicenter study would help to get accurate data for clarification.

Most importantly, specifically under the different photopic test conditions and in the resting state condition, a significant benefit of the selective blue-filtering lens over the placebo lens could be shown, representing essential domains of daily living and thus having a substantial impact on the quality of life of people with BEB. This is supported by another study, showing increased blink frequency with light stimuli which decreased with 590 nm and 480 nm blocking lenses (15). A possible explanation for the improvement of BEB under different photopic test conditions, while wearing blue-filtering spectacles, is the evidence of greater photosensitivity in BEB compared to healthy controls (15).

The limitation of this study is the small sample size, albeit keeping in mind that this is a rare disease (prevalence 16–133 cases per million) (16). In addition, this study did not use a 2 weeks wash-out procedure between the two spectacles, as in the study by Blackburn et al., and it used a retrospective manual count of blink frequency by two independent raters rather than a surface electromyogram, as in the study by Blackburn et al., as a potential methodological bias (13).

In conclusion, these glasses might provide a potential benefit over non-optical glasses and could be a new non-invasive therapeutic add-on option, in addition to BoNT therapy, for patients with BEB.

Hence, non-invasive therapeutic concepts such as photochromic blue filter glasses should be necessarily further investigated in a multicenter setting resulting in larger sample sizes and therefore gaining valid information, especially considering the potentially high impact on quality of life. Subsequently, it would be important to implement patient-reported outcomes regarding subjective improvement of blink frequency, spasms, photophobia, and ultimately quality of life.

## Data availability statement

The raw data supporting the conclusions of this article will be made available by the authors, without undue reservation.

## Ethics statement

The studies involving humans were approved by Ethics Committee of the Medical University of Vienna. The studies were conducted in accordance with the local legislation and institutional requirements. The participants provided their written informed consent to participate in this study.

## Author contributions

TM: Data curation, Formal analysis, Investigation, Methodology, Project administration, Writing – original draft. TZ: Data curation, Formal analysis, Investigation, Methodology, Project administration, Writing – original draft. CW: Conceptualization, Data curation, Investigation, Methodology, Project administration, Writing – original draft. ZK: Data curation, Formal analysis, Resources, Supervision, Validation, Writing – review & editing. WG: Data curation, Formal analysis, Resources, Supervision, Validation, Writing – review & editing. TS: Conceptualization, Data curation, Formal analysis, Funding acquisition, Methodology, Project administration, Resources, Supervision, Validation, Writing – review & editing.
